# The challenges of using ultrasound to measure the trachea: a brief report

**DOI:** 10.1186/s13054-025-05499-0

**Published:** 2025-07-01

**Authors:** Helen Newman, Jodi Allen, Madan Narayanan, Nchafatso Obonyo, Nur Syahrunnizar, Karen Hammet, David Parry, Sarah Wallace, Anna-Liisa Sutt, Joseph Osterwalder, Daniel Martin

**Affiliations:** 1 Division of Surgery and Interventional Science, University College London, Royal Free Hospital, 3rd Floor, Pond Street, London, NW3 2QG UK; 2 Barnet Hospital, Royal Free London NHS Foundation Trust, Wellhouse Lane, Barnet, EN5 3DJ UK; 3 The National Hospital for Neurology and Neurosurgery, University College London NHS Foundation Trust, London, UK; 4 University College London Centre for Medical Imaging, London, W1W 7TS UK; 5 Frimley Park Hospital, Frimley Health NHS Foundation Trust, Frimley, GU16 7UJ UK; 6 Critical Care Research Group, The Prince Charles Hospital, Brisbane, Australia; 7 Institute for Molecular Bioscience, The University of Queensland, Brisbane, Australia; 8 IDeAL, KEMRI-Wellcome Trust Research Programme, Kilifi, Kenya; 9 Wellcome Trust Centre for Global Health Research, Imperial College London, London, UK; 10 Department of Anatomy, King’s College London, London, SE1 1UL UK; 11 Department of Speech Voice and Swallowing, Wythenshawe Hospital, Manchester University NHS Foundation Trust, Southmoor Road, Manchester, M23 9LT UK; 12 Division of Infection Immunity and Respiratory Medicine, School of Biological Sciences, Faculty of Biology Medicine and Health, University of Manchester, Oxford Road, Manchester, M13 9PL UK; 13 Barts Health NHS Trust, London, UK; 14 Polipraxis, St. Gallen, 9000 Switzerland; 15 Peninsula Medical School, University of Plymouth, John Bull Building, Plymouth, Devon, PL6 8BU UK

**Keywords:** Tracheostomy, Ultrasound, Trachea, Sonoanatomy

## Abstract

**Background:**

Tracheostomy is a common procedure in intensive care medicine, involving the insertion of an artificial airway through the front of the neck into the trachea. The size of tracheostomy tube in relation to the trachea is important, influencing clinical outcomes and patient experience. Ultrasound is readily available at the bedside and airway applications have been reported, including airway measurement. However, the presence of air within airway lumina presents challenges to ultrasound and causes artefact. Guidance is lacking on tracheal sonography and measurement.

**Aim:**

to characterize the sonoanatomy of the inner tracheal wall and determine reference points for measuring tracheal diameter.

**Methods:**

exploratory study; pig, sheep and human tracheas were flooded to eliminate air artefact. Still and video images of flooded and dry specimens were compared to help differentiate air artefact from anatomical landmarks; locate the inner tracheal wall; and determine reference points for measurement.

**Results:**

The inner tracheal wall presented as a bright line anteriorly but was not visible where cartilages were calcified or laterally. Mirror images of the tracheal wall and peri-tracheal structures were seen within the air column. A method of measuring the trachea was developed based on the outer border of the trachea and its reflection.

**Conclusion:**

Tracheal rings can be identified by distinctive sonographic features. Inner tracheal width may be estimated by averaging the diameters of the outer-tracheal border and its reflection within the air column. Further clinical studies of relevant patient populations are needed to determine accuracy and reliability of estimates.

**Supplementary Information:**

The online version contains supplementary material available at 10.1186/s13054-025-05499-0.

## Introduction

Tracheostomy is a common procedure in intensive care medicine, involving the insertion of an artificial airway through the front of the neck into the trachea. Common indications are the need for prolonged mechanical ventilation and to provide a safe airway in cases of airway obstruction [1, 2]. The size of tracheostomy tube is important. It should be large enough to allow effective breathing, mechanical ventilation and tracheal suctioning through the tube, but small enough to avoid tracheal trauma and allow use of a one-way valve to restore important laryngeal functions such as airway protection, voicing, coughing and physiological regulation of end expiratory pressure [1, 3]. Incorrectly sized tubes impact these functions, may be displaced and can lead to tracheal stenosis [4, 5]. Little guidance exists on tracheal imaging in radiology or head and neck anatomy textbooks and there is no reference-standard tracheal measurement technique. Reported airway ultrasound applications are increasing and include: confirming endotracheal tube placement; screening for blood vessels; measuring airway diameter; and aiding selection of airway-tube size [6–8]. Ultrasound is non-invasive and readily available at the bedside, however, airway ultrasound can be challenging due to the presence of air within the lumina, which is a poor conductor of soundwaves and causes image artefact [9]. A few studies have found ultrasound measurements of the sub-glottic/cricoid space correlate well with MRI and CT, however contradictions exist in the interpretation of anatomy and artefact [6, 10, 11]. To date we lack a reliable description of the sonoanatomy of the inner tracheal wall and guidance on identifying reference landmarks to use in measurement. The aims of this exploratory study were to characterize the sonoanatomy of the inner tracheal wall and determine reference points for measuring tracheal diameter.

## Methods and materials

We conducted exploratory observational work, collecting still and video ultrasound images of pig, sheep and cadaver tracheas. Porcine and ovine airway models have previously been used in medical research due to their similar size and form to human anatomy [12–14]. Table 1 shows the type of specimen, source, views obtained, examiner, and equipment used in this study. The pig trachea was provided by a local butcher; sheep had been part of a trial investigating septic shock resuscitation, and the fresh cadaver was being used for research and education in a university anatomy department.

All specimens were scanned using high frequency linear probes, which are appropriate for superficial structures. Machine settings were selected based on image clarity, commencing with manufacturer presets for imaging superficial structures and tissue harmonic imaging switched on, adjusting other settings (depth, dynamic range and gain), as required. Expert opinion on technical settings and image interpretation was provided by a head and neck sonographer and consultant radiologist.

In the cadaver, the thyroid cartilage notch was identified visually and through palpation. A single-frame anterior longitudinal view from thyroid cartilage to trachea was captured first to confirm position of the thyroid, cricoid and tracheal cartilages. A descending sweep of the trachea was performed in the midline from the thyroid cartilage to the sternal notch with the transducer angled at 90 degrees to the trachea. Similar imaging was also obtained from one of the investigators (HN).

Unlike air, water is a good conductor of soundwaves. We therefore flooded the trachea specimens with water to facilitate imaging the airway and allow anatomical landmarking of the inner tracheal wall in the absence of air-artefact. Landmarking was then mapped onto images of the same specimen and view in the presence of air. Air artefact was described and compared to artefact seen in tracheal ultrasound images from a related observational study (unpublished), and a method for obtaining tracheal width measurements was developed.

A related study investigating measurement accuracy and reliability in three blinded reviewers’ measurements of six fresh-frozen cadavers was unfortunately unsuccessful due to a lack of adequate gold-standard, direct measurement methods - CT and MRI were not available and future embalmment prohibited dissection for direct visualization (unpublished).

Existing ethical approval and tissue storage licenses were in place for the use of the sheep and cadaver (see Ethics below). No approvals were needed for the pig specimen as it was a bi-product of the food industry.

**Table 1:** Specimens, sources, equipment and images collected

Specimen/subject	Source	Equipment	Examiner	Images
Pig (resected trachea)	Local butcher - bi-product of food industry	GE Venue™ POCUS machine (Chicago, US)	MN, consultant intensivist, lead trainer in POCUS	Dry, water-flooded, transverse
Sheep (full specimen, imaged in side-lying)	Critical Care Research Group, Brisbane – ex-vivo, part of trial investigating septic shock resuscitation	GE Venue™ POCUS machine (Chicago, US)	NO, Postdoctoral research fellow	Dry, water-flooded, transverse and longitudinal
Human cadaver (full specimen)	King’s College London Dissecting Rooms – non-embalmed, non-frozen specimen	Mindray TE7™ POCUS machine (Huntingdon, UK)	HN, clinical specialist SLT, PhD candidate	Dry, water-flooded, transverse and longitudinal
First author, HN	n/a	Mindray TE7™ POCUS machine (Huntingdon, UK) Collected same day as cadaver study images.	HN, clinical specialist SLT, PhD candidate	Dry, transverse, longitudinal

POCUS = point of care ultrasound

## Results

### Characterization of tracheal sonoanatomy

**Fig. 1:**
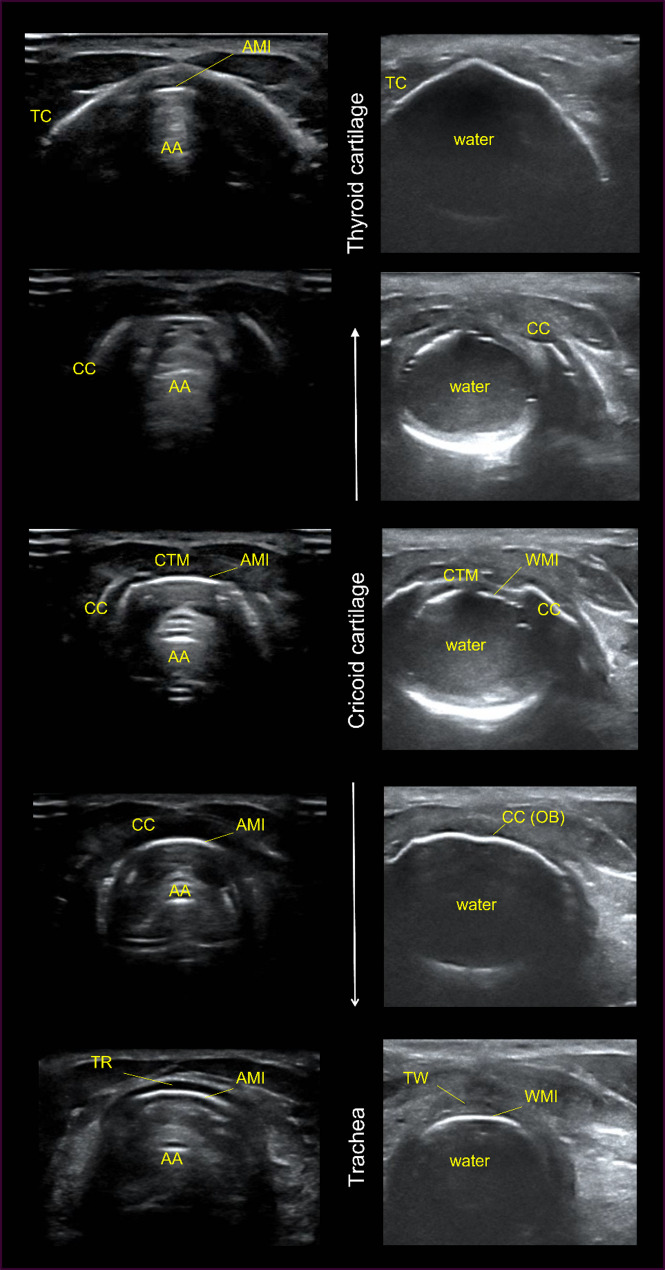
Changing form of the cricoid cartilage on descending transverse view from the thyroid cartilage to first tracheal ring (or soft tissue above). Left – volunteer (air-filled), right – cadaver (water-filled). TC = thyroid cartilage; AA = air artefact; AMI = air-mucosa interface; CC = cricoid cartilage; CTM = cricothyroid membrane; WMI = water-mucosa membrane; CC (OB) = cricoid cartilage (outer border); TR = tracheal ring; TW = tracheal wall between rings

Tracheal rings were easily distinguished from the cricoid cartilage in longitudinal views of the cadaver and volunteer: the cricoid was thicker and sat between the thyroid cartilage and first tracheal ring. On descending transverse sweeps in the midline, the profile of the cricoid changed, with only the sides appearing on higher views, separated by the cricothyroid membrane (Fig. 1). These progressively approached to meet in the midline on lower views. The tracheal rings were thinner, more uniform in profile, and further differentiated by the presence of thyroid tissue anteriorly and laterally.

Calcified cartilages in the cadaver created shadowing or blackout of the image below them. The air-mucosa interface (AMI) and air artefact in the cadaver trachea were therefore not visible behind tracheal cartilage. Images were obtained by positioning the transducer in between cartilages and scanning through soft-tissue segments of the tracheal wall instead.

**Fig. 2:**
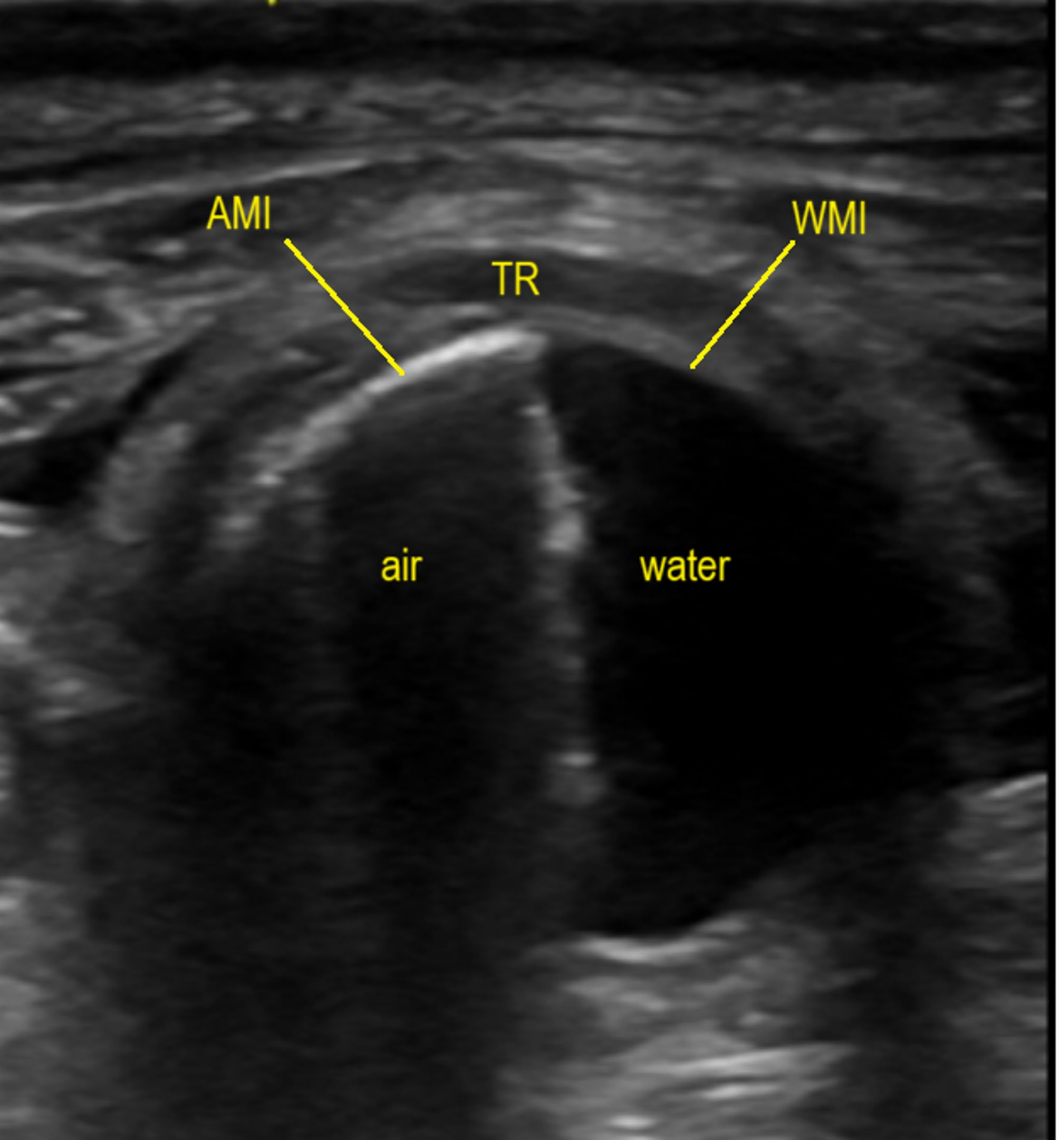
Hemi-flooded sheep trachea, aiding identification of the air-mucosa interface through location in relation to the water-mucosa interface. TR = tracheal ring; AMI = air-mucosa interface; WMI = water-mucosa interface

The inner tracheal wall was identified in the flooded sheep and cadaver tracheas. The corresponding area, or AMI, was then identified on matching views of the same specimen with air present. Images of the half-flooded sheep trachea with the sheep in side-lying permitted comparison of air and water-filled trachea within the same frame (Fig. 2). Ultrasound signal within the tracheal lumen on the air-filled portion was identified as artefact. The introduction and expulsion of a pocket of air within the cadaver trachea permitted video footage of the appearance and disappearance of air-artefact. Lines in air-filled frames that were not present in flooded frames were interpreted as artefact. The AMI was brighter than lines of artefact. In the volunteer, the soft tissue between tracheal rings appeared less dark than tracheal ring cartilage and the adjacent AMI appeared less bright in both transverse and longitudinal views.

**Fig. 3:**
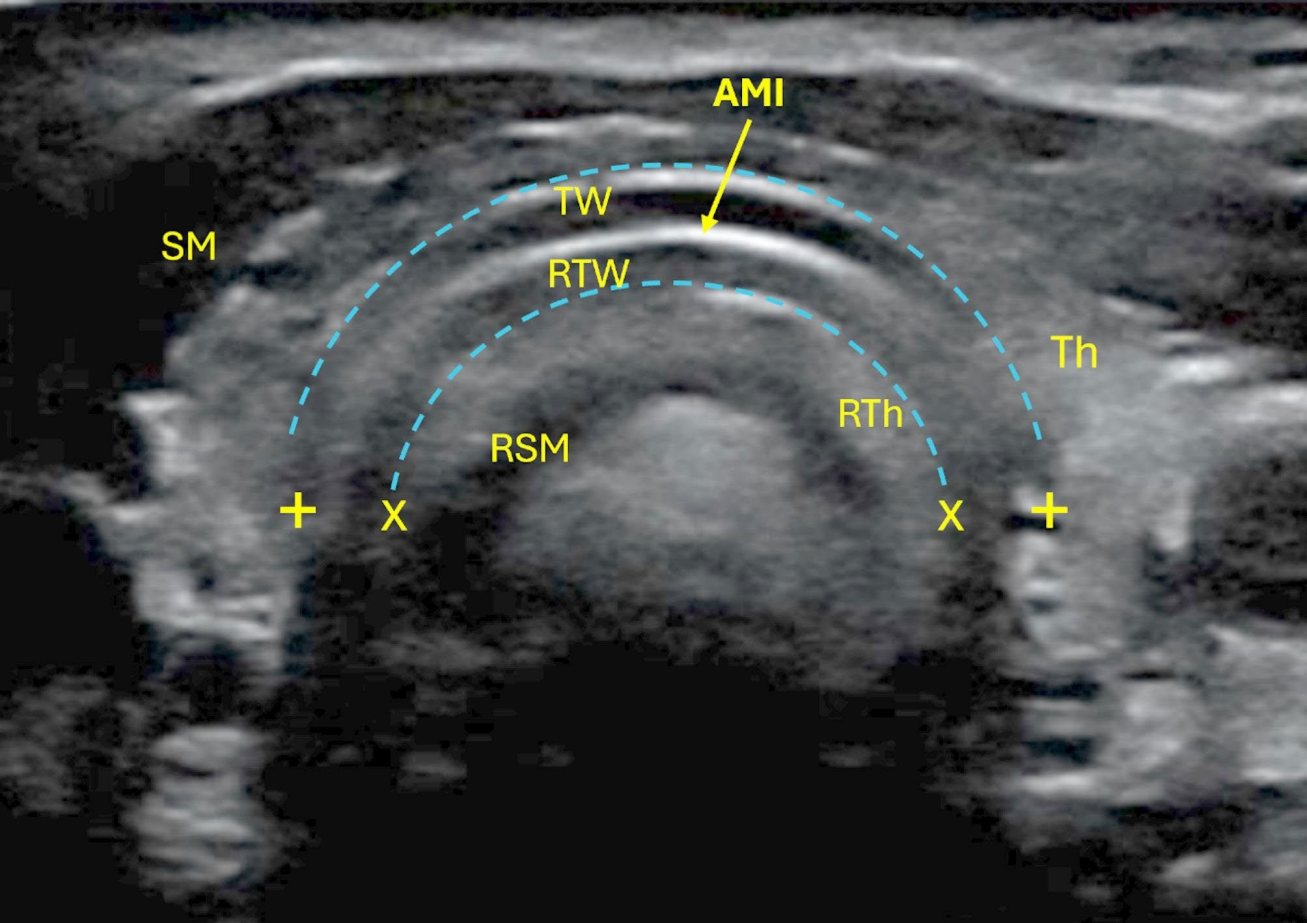
Mirror artefact within the air column. SM = strap muscles; RSM = reflected strap muscle; TW = tracheal wall; RTW = reflected tracheal wall; AMI = air-mucosa interface; RTh = reflected thyroid; ‘+’ caliper – outer tracheal border; ‘x’ caliper – reflected outer tracheal border

In the volunteer and sheep, air artefact created mirror-images of the tracheal wall within the air column which were more prominent at tracheal rings than soft tissue portions. Reflected thyroid tissue and muscle could also be seen in the volunteer as shown in Fig. 3 (see Additional File 1 for more examples).

### Method for measuring the trachea

**Fig. 4:**
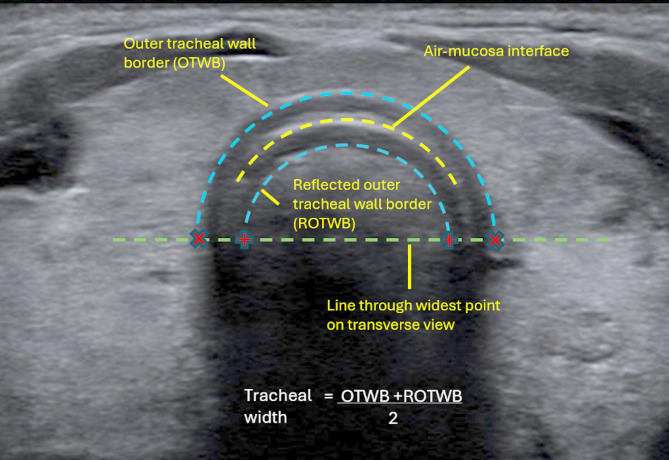
Image interpretation and reference measurement points. Red ‘+’ and ‘x’ calipers indicate diameters of outer tracheal wall border and its reflection, respectively

The AMI did not extend to the widest point of the trachea on any images and could not, therefore, be used for obtaining tracheal width measurements. The lines representing the outer border of the tracheal wall and its reflection in the air column extended further laterally than the AMI and by extrapolating slightly their diameters at the widest point could be measured. As one was a reflection of the other around AMI, the mean of the two diameters was used as an estimate for tracheal width (see Fig. 4).

## Discussion

We used a novel approach to eradicate air artefact from tracheal sonograms, allowing us to characterise tracheal sonoanatomy and artefacts, and propose a new method for obtaining tracheal width measurements. Such measurements could improve clinical outcomes and lived experiences of critically ill patients through better selection of tracheostomy tube size. Tracheal rings were distinguished from the cricoid cartilage by their smaller thickness, uniform transverse profile, and position in relation to other structures. We found that the AMI was the brightest line of the airway wall, except in cases of calcified cartilages. Here the brightest white line represented the outer border of the airway wall and the AMI was not visible. Mirror artefact caused reflection of the tracheal wall and peri-tracheal structures within the air column. A tracheal-measurement method was developed based on averaging the diameters of the outer tracheal wall and its reflection within the air column.

Other authors have interpreted tracheal imaging differently. Lakhal et al. (2007) present a figure labelled the cricoid cartilage [6]. However, it shows a thin cartilage surrounded by mid-greyscale tissue, suggesting it is a tracheal ring surrounded by thyroid tissue. Moreover, in measuring airway diameter, the calipers appear to be placed on what we showed to be the reflection of the outer tracheal border, as others have done also [10, 15, 16], which would lead to an underestimation of tracheal width. Lakhal et al. also describe the AMI as ‘hypoechoic’ (dark) [6]. Our finding that the AMI was the brightest line fits with evidence from the radiology literature that states that interfaces between objects with strongly contrasting echogenicity appear ‘*hyper*echoic’ (bright) [9, 11]. Tsui, Ip, and Walji’s (2013) study concurs with our description of the changing sonographic appearance of the cricoid from superior to inferior borders [17].

Previous studies of ultrasound-aided tracheal measurement have used comparison with reference diagrams, alternative imaging techniques, or findings in cadaveric specimens to justify caliper placement, while others have provided no support. Few have discussed artefact [10, 15, 18]. We controlled imaging conditions, comparing flooded with air-filled imaging of the same trachea to provide empirical support for our anatomical landmarking. We were unable to use the AMI line for measurements as it was not visible at the lateral inner tracheal wall. This is likely because creation of the bright line requires reflective interfaces to be perpendicular to the ultrasound beam.

There is little scientific evidence on mirror artefact in the trachea. Radiology descriptions of mirror artefact usually relate to the gallbladder reflected around the diaphragm, and airway artefact is usually presented as repeating lines of reverberation [11]. However, You et al. (2018) report a case of a thyroid cyst mirrored within the tracheal lumen and mirror artefact is discussed in a review of airway ultrasound in the radiology literature [19, 20]. Similar mirror artefact was observed in most of the images collected in an observational study related to the work presented here (unpublished). Recognition of mirror artefact was important, since erroneously interpreting mirrored anatomy as a true landmark could lead to underestimating the size of the trachea and/or poor measurement reliability. It also enabled the development of our sonographic method of measuring tracheal diameter. The observation that calcified cartilages obscure deeper structures and mirror-artefact suggests this measurement method may be less successful in older patients.

Our findings suggest that, contrary to some claims, airway ultrasound is challenging and requires good understanding of ultrasound physics as well as airway anatomy. This should be reflected in training programs for ultrasound airway measurement.

## Limitations

This small study relied on cadaver and animal data, impacting generalizability to the clinical setting. However, by using fresh and fresh-frozen cadavers, we avoided the impacts of embalming or freezing on tissue echogenicity, and the younger, more echoic tissues of the animal specimens permitted clearer imaging of the AMI. Endotracheal tubes were not present during imaging, which may impact generalizability to the intensive care setting. Edge artefact at the outer tracheal border meant caliper placement relied on examiner judgement, as did extrapolation of the reflected outer tracheal border. Variation in equipment and examiner experience are also acknowledged. Inter- and intra-observer variability were not assessed in this exploratory study and remain limitations to be addressed in future work. Further, as previously mentioned, attempts to determine measurement accuracy in a related study were unsuccessful due to lack of suitable ‘gold-standard’ comparator. Lastly, tissue harmonic imaging was used during data collection which is reported to attenuate reverberation artefact [21] and may explain the occurrence of mirror artefact and reduction in reverberation artefact in this work.

## Conclusions

Tracheal rings can be differentiated from the cricoid cartilage by their reduced thickness, uniform profile, and inferior vertical position. The inner tracheal wall presents as a distinctive bright AMI line except where cartilages are calcified, in which case it cannot be seen. Mirror images of structures outside the inner tracheal wall can be seen in the air column and may be machine-setting dependent. The averaged diameter of the outer tracheal border and its reflection within the air column provides an estimate of the inner tracheal diameter. Feasibility, accuracy, and reliability testing of this measurement method should be conducted in relevant patient populations before widespread adoption in clinical practice and will depend on identifying suitable reference measurement techniques with which to compare.

## Electronic supplementary material

Supplementary Material 1

## Data Availability

Data is provided within the manuscript.

## Abbreviations

POCUS: Point of care ultrasound
AMI: Air-mucosa interface
WMI: Water-mucosa interface
