# Supplementary material for: The challenges of using ultrasound to measure the trachea: a brief report
Source: Crit Care. 2025 Jul 1;29:265. doi: 10.1186/s13054-025-05499-0 (PMC12219974; doi:10.1186/s13054-025-05499-0)
Supplement: Supplementary file 1 — Supplementary Material 1 [file 13054_2025_5499_MOESM1_ESM.docx]

**Additional File 1**

**Differentiating mirror artefact from anatomical structures on tracheal ultrasound**

The clinician scanning the trachea should have a good understanding of the anatomy of the trachea and surrounding structures, including relative size and position. The cricoid cartilage is thicker than the tracheal rings and lies cephalad to them. It is further distinguished by the absence of adjacent thyroid tissue.

Figure 1. below shows a transverse ultrasound view of a tracheal ring. At the top of the image lies the skin of the anterior neck. The bright white arch in the center top third is the air-mucosa interface (AMI) at the surface of the inner tracheal wall, which acts as a reflective plane. Above and laterally to the AMI are true anatomical structures and all shapes beneath it are air-related artefact. Strap muscle, thyroid tissue, and a tracheal ring are highlighted on the left of the image, with matching colors used to highlight their reflections within the air column.


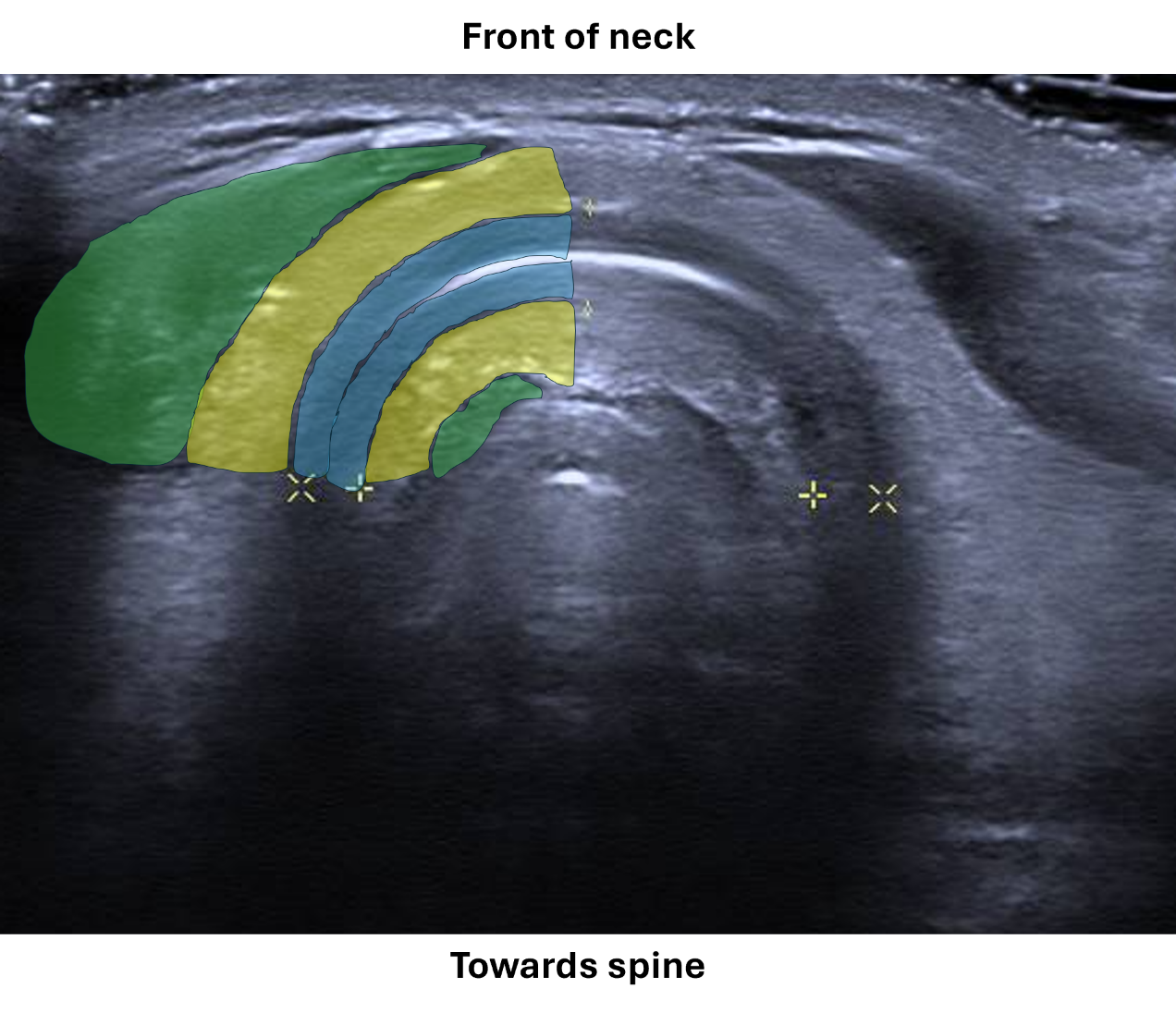


Figure 1. Anatomical structures and mirror artefact. Green = strap muscle and reflected strap muscle; yellow = thyroid tissue and reflected thyroid tissue; blue = tracheal ring and reflected tracheal ring. Paired calipers mark the outer border of the tracheal wall (x) and its reflection within the air column (+) at the full width of the trachea.
